# Dilemmas in the Management of Liminal Rodents—Attitudes of Dutch Pest Controllers

**DOI:** 10.3390/ani10091614

**Published:** 2020-09-09

**Authors:** Maite A.A.M. van Gerwen, Joachim Nieuwland, Hein A. van Lith, Franck L.B. Meijboom

**Affiliations:** 1 Centre for Sustainable Animal Stewardship (CenSAS), Department Population Health Sciences, Faculty of Veterinary Medicine, Utrecht University, Yalelaan 2, 3584 CM Utrecht, The Netherlands; j.nieuwland@uu.nl (J.N.); f.l.b.meijboom@uu.nl (F.L.B.M.); 2 Section Laboratory Animal Science/3Rs-Centre, Unit Animals in Science and Society, Department Population Health Sciences, Faculty of Veterinary Medicine, Utrecht University, Yalelaan 2, 3584 CM Utrecht, The Netherlands; h.a.vanlith@uu.nl; 3 Brain Centre Rudolf Magnus, University Medical Centre Utrecht, Universiteitsweg 100, 3584 CG Utrecht, The Netherlands; 4 Ethics Institute, Faculty of Humanities, Utrecht University, Janskerkhof 13, 3512 BL Utrecht, The Netherlands

**Keywords:** animal ethics, animal welfare, commensal rodents, liminal rodents, pest control, rodent control, rodent management

## Abstract

**Simple Summary:**

Most people think the welfare of non-human animals matters. However, when it comes to rats and mice labeled as ‘pests’, welfare generally appears less important. Together with stakeholders in the field of pest management, we are working to develop a framework for less harmful rodent control that can be used by pest controllers. An online survey was carried out in order to find out to what extent Dutch pest controllers take the welfare of rats and mice into account as part of their profession. Our findings show that respondents pay attention to animal welfare and believe that some methods used cause severe animal suffering. Also, they think there are situations in which more attention for preventive methods (e.g., cleaning, removing food sources, or closing holes in a building) benefits both humans and pest animals. They indicate, however, that it is sometimes hard to include animal welfare in their work. An important reason for this is that clients do not always want to invest sufficient money in prevention. The findings of this study are useful for further conversations with pest controllers and their clients on how to safeguard animal welfare. Furthermore, they are relevant to the framework we are developing.

**Abstract:**

When non-human animals are labeled as ‘pests’, their moral status and welfare seem relatively unimportant. In a multi-stakeholder project, we develop an assessment frame for a more responsible rodent management that includes animal welfare. An online survey among 129 Dutch pest controllers was carried out in order to find out more about pest controllers’ attitudes about animal welfare. Respondents indicate to consider animal welfare in their job. They see differences in the welfare impact of different rodent control methods. A dilemma may occur when methods with a high impact, such as rodenticides, are ofttimes used in practice. Respondents also indicate that in different real-life scenarios (the hospital kitchen vs. the private backyard), a different weight may be attributed to the importance of animal welfare. Almost half of the respondents encounter difficulties when weighing animals against human interests. The problems are mainly related to clients who are not willing to invest sufficient money in preventive methods, where respondents do believe in. Some differences were found between respondents depending on membership of a professional association for pest controllers. The results of this study are relevant input for focus groups with pest controllers and their clients and for the development of the aforementioned assessment frame.

## 1. Introduction

Discussions about the treatment and welfare of non-human animals (hereafter ‘animals’) usually concern livestock, companion animals, laboratory animals, or large wild animals. However, the range of human-animal interactions includes many more animals. These animals are neither wild, nor domesticated. Living their lives amidst humans, between nature, and culture, these animals, such as commensal rats, house mice, and pigeons can be considered *liminal* [1]. For the purpose of, e.g., food safety, human and animal health, hygiene, and safety of stables, all over the world, unspecified but large numbers of rats (*Rattus norvegicus* [2] and *Rattus rattus* [3]) and house mice (*Mus musculus* [3]) are killed (with several methods inflicting significant levels of suffering [4]) because they are perceived as ‘pests’ or ‘vermin’. It is striking that once labeled as a pest animal, discussions on moral status and welfare seem to disappear from both the scientific and public debate. In the relatively small amount of papers on the treatment of these animals, authors point out the inconsistency in the treatment of different animals depending on the context [5,6]. For instance, within the current legislation in most Western societies, people have comparatively a lot of freedom to decide how to deal with these liminal rodents that do not differ in their capacities to suffer compared to rats and mice in other practices, such as the laboratory or as pets [7]. In scientific research for the production of rat poison, a rat will be monitored and treated in accordance with humane endpoints (A humane endpoint can be defined as: “the earliest indicator in an animal experiment of (potential) pain and distress that, within the context of moral justification and scientific endpoints to be met, can be used to avoid or limit the pain and distress by taking actions such as humane killing or terminating, or alleviating the pain and distress” [8]) to prevent unnecessary suffering. A rat perceived as a pest animal often dies a slow and painful death having ingested the same poison [6]. This inconsistency generally remains in the background, as pest management is largely outsourced to professionals rendering the used control measures a black box [6,7,9].

In order to facilitate ethical decision-making and include the moral status and welfare of liminal rodents in pest management, various authors have previously indicated animal research ethics as a valuable source. This includes attention for animal welfare (e.g., minimizing suffering), as well as the specific way of justifying actions by means of a decision-making framework [6,7,10,11,12]. Whereas in the research context, animals are subjected to a wide range of different procedures that inflict suffering, requiring ethical assessment, in pest management, the focus lies with the method of killing primarily. The welfare implications of pest control methods are usually described by the amount of pain and distress, the duration, the methods for killing, and the effects on animals that either escape from the control or non-target species [4,5,6,7]. Broom [4] has classified different control methods by means of their humaneness. Fast-acting killing traps are classified as humane, whereas slow-acting anticoagulants (one of the most used control methods in the Netherlands and most other Western societies) are classified as inhumane [4,5] or severe suffering [12].

### 1.1. Integrated Pest Management

In the Netherlands, since 2017, the use of anticoagulant rodenticides requires a certification for Integrated Pest Management (IPM) for the control of rats outside buildings [13]. According to the IPM principles, preventive and non-chemical methods (e.g., mechanical killing traps) should be used before rodenticides [see 6 for a description of IPM]. Preventive methods can consist of cleaning the area, removing food sources, closing buildings, etc. While animal welfare appears a strong argument to consider such a requirement, the primary motivation derives instead from environmental concerns and the increasing resistance in rats against the poison. Nevertheless, the correct application of the IPM principles with a focus on prevention benefits the interests of liminal rodents as well.

### 1.2. Stakeholder Views

In 2018 the Centre for Sustainable Animal Stewardship (CenSAS) performed a stakeholder consultation regarding the treatment of rats and mice perceived as pests [14]. Stakeholders (17 persons, all Dutch) represented local and national government(s), animal protection NGOs (Non-governmental organizations), pest controllers, food retail, agricultural sector, or were researchers or advisors in the field of pest control and wildlife management. The consultation highlighted a shared need to take the moral status and welfare of pest rodents more seriously. Everyone supported the need for better application of preventive methods both by professionals and citizens. Control measures should be categorized in terms of suffering, and their usage justified based on clearly stated conditions. Finally, to better implement IPM and responsible rodent management, national coordination, and monitoring under the responsibility of the government is considered crucial.

The outcomes of this earlier study were the start of a multi-stakeholder project, initiated by, and under the lead of CenSAS, for an assessment frame that contributes to more responsible rodent management in which moral status and welfare of rodents are considered. In order to identify relevant moral concerns and dilemmas, as well as to develop an adequate frame that will support ethical decision-making in practice, the knowledge and experience of pest management professionals are indispensable. An online survey among Dutch pest controllers was, therefore, carried out in order to find out more about their attitudes regarding animal welfare in rodent control, their experiences of (moral) dilemmas in practice, and possible ways (among which the use of an assessment frame) to overcome these dilemmas.

## 2. Materials and Methods

### 2.1. Survey

The online survey (details of the survey are available from the corresponding author upon demand) was set up in Dutch using Google Forms. The answers were translated into English for the purpose of this article. The link to the survey, together with the call to take part, was placed on the website of CenSAS and spread via professional associations of pest controllers, newsletters, and websites of stakeholder organizations, personal networks, and social media. No sample size had been predefined since the main function of the survey was to provide a descriptive overview of views and opinions. The survey was open during April and May 2019. The expected time to complete the survey was 15 to 20 min. The results were obtained and processed anonymously. Questions (37 in total) in the survey were of different types using five-point Likert-style questions (never, seldom, sometimes, often, always), 10-response (equally spaced) interval rating scales from 1 (e.g., totally disagree or not important) to 10 (e.g., totally agree or very important), open, multiple-choice, and multiple response questions. A range from 1 to 10 was used for the rating scales since this is a familiar range for Dutch people due to its use for grading at schools. For some questions, respondents had the possibility to provide additional information or add additional answers not provided in the lists. At the beginning of the survey, rats and mice were specified as black and brown rats (*Rattus rattus* and *Rattus norvegicus*, respectively) and house mice (*Mus musculus*).

The survey was divided into six sections. The first section consisted of five statements about the general perception of rats and mice, where respondents could agree with or not on a 1 (totally disagree) to 10 (totally agree) rating scale.

The second section about animal welfare consisted of six questions about the (relative) importance of rodent welfare (Likert scales and 1 to 10 rating scales), conceptions of animal welfare (multiple-response questions), the impact of 10 control methods in terms of animal suffering on a 1 (no welfare impact) to 10 (very large welfare impact) rating scale, and the motivation to improve rodent welfare in pest management on a 1 (I do not want to do more) to 10 (I want to do a lot more) scale. As a further specification, animal interests were defined as ‘living, freedom, and welfare’.

The third section consisted of questions about the type of clients (type of business, sector, etc.), the amount of awareness among clients (Likert scales), and the client’s willingness to invest in preventive methods on a 1 (no willingness) to 10 (much willingness) scale.

The fourth section was to get insight into the attitudes towards existing regulations, IPM, and the belief in prevention. The section consisted of three statements about regulations and IPM. It contained one open question about prevention where respondents were asked to indicate what percentage of nuisance could be solved by preventive methods only according to them.

The fifth section about experience and decisions in daily practice consisted of questions about important reasons to work as a pest controller on a 1 (very unimportant) to 10 (very important) rating scale, the weight of (or space for) animal interests in 12 different real life scenarios on a 1 (animal interests do not count) to 10 (animal interests count heavily) scale, the experience of problems or dilemmas (multiple choice and open questions), and the possible solutions for these problems on a 1 (no added value for solving problems) to 10 (large added value for solving problems) scale.

At the end of the survey (sixth section), several demographic data were collected, such as gender, age, membership of a professional association for pest controllers, type of employment, and work region. Furthermore, pest controllers were asked if they had taken any post-graduate course in ethics for pest controllers. Membership of an association for pest controllers could be indicated on four levels: a member of NVPB (Nederlandse Vereniging Plaagdiermanagement Bedrijven), member of PLA..N. (Platform Plaagdierbeheersing Nederland), a member without specification of the association (unspecified member), no member.

### 2.2. Statistical Analysis

Analysis of the survey results was done with the IBM^®^ SPSS^®^ Statistics for Mac (Version 26) computer program (IBM Corp., Armonk, NY, USA), using descriptive statistics and inferential statistics for the interval rating data. For this type of data, the Friedman repeated measures test (*omnibus*) and the Wilcoxon matched-pairs signed-ranks test (*post-hoc*) were used to test for differences between dependent variables (e.g., ‘scored welfare impact of different control methods’ and ‘weight of animal interests in different practical scenarios’). The Kruskal–Wallis test (*omnibus*) and Wilcoxon–Mann–Whitney test (*post-hoc*) were used for testing differences between independent grouping variables (factors). The following grouping variables were tested: (1) with or without post-graduate ethics education, (2) membership of an association for pest controllers, and (3) ownership of companion animals. Descriptive statistics of interval rating data (on a 1 to 10 scale) were provided as medians with interquartile ranges (IQR) and displayed in box plots (also known as box-and-whisker plots). Box plots show median values with IQR, highest and lowest non-outlying values (i.e., values up to 1.5 box lengths from the upper or lower edge of the box). In the figures with the box plots (mild) outliers (i.e., cases with values between 1.5 and three box lengths from the upper or lower edge of the box) and extreme cases (i.e., cases with values more than three box lengths from the upper or lower edge of the box) are also indicated.

For the *omnibus* and *post-hoc* tests Monte Carlo (number of samples was 10,000) and exact *p* values (two-tailed) were respectively calculated. To compensate for the increased chance of a type I error as a consequence of multiple hypotheses testing, values of *alpha* (*α*) were adjusted with the Dunn–Šidák correction. The formula for calculating the *adjusted alpha* was: *α_adj_* = 1 − [1 − *α*]^1/*γ*^; in which *γ* is the number of hypotheses tested (*omnibus* tests: ‘number of test variables’ multiplied by ‘the number of *omnibus* tests performed’; *post-hoc* tests: ‘number of test variables’ multiplied by ‘the number of *omnibus* tests performed’ multiplied by ‘the number of pairwise comparisons’) per topic, and *α* = 0.05. The adjusted *alpha* values for each test used can be found in Supplementary Table S1. Significant *p* values are marked with an asterisk (*) and are reported with six (e.g., *p* = 0.000414*) or seven (*p* < 0.0000005*) decimal places, whereas non-significant *p* values are reported with two (e.g., *p* = 0.16) to six digits (e.g., *p* = 0.00009) after the decimal point.

Statistical significance represented by *p* values may not necessarily confirm practical importance. In our opinion, the size of the observed effects is perhaps more important than statistical significance. Therefore, besides *p* values, estimated effect sizes were calculated for both the *omnibus* and *post-hoc* tests. Effect sizes reported comprise (1) Kendall’s *W* value for the Friedman repeated measures test, (2) the eta squared value (*η^2^*) for the Kruskal–Wallis test, and (3) the correlation coefficient *r* for the Wilcoxon matched-pairs signed-ranks test, and the Wilcoxon–Mann–Whitney test. The formulas [15] are as follows:

(1) $$W=\frac{\chi^{2}}{n\left(k-1\right)}$$

(2) $$\eta^{2}=\frac{H-g+1}{n-g}$$

(3) $$r=\frac{Z}{\sqrt{n}}$$

where ‘*n*’ is the number of observations (2 × 129 in case of the Wilcoxon matched-pairs signed-ranks test), *k* is the number of repeated variables per topic, *χ*^2^ is the chi-square statistic for the Friedman repeated measures test, *H* is the chi-square statistic for the Kruskal–Wallis test, *g* is the number of groups per factor, and *Z* is the standardized *z*-score of the Wilcoxon matched-pairs signed-ranks test or the Wilcoxon–Mann–Whitney test. The thresholds used for qualitative descriptions of the (absolute) values of effect size (i.e., *W*, *η*^2^, or |*r*|) are shown in Table 1. Exact *p* values and absolute effect sizes for Wilcoxon matched-pairs signed-ranks test (*post-hoc*) could be found in the Supplementary Tables S2–S9. The exact *p* values and absolute effect sizes for the Wilcoxon–Mann–Whitney test (*post-hoc*) can be found in the Supplementary Table S10.

### 2.3. Ethical Approval

For this type of research, no ethical approval is required in the Netherlands. The design and analysis of the survey are in accordance with the code of conduct of the Association of the Universities in the Netherlands and the General Data Protection Regulation (GDPR) of Utrecht University.

## 3. Results

### 3.1. Demographics

The online survey was completed by 129 Dutch pest controllers (the respondents from now on), mostly male (93.8%). Most respondents (41.9%) were between 41 and 50 years or between 51 and 67 (Dutch retirement age) years (31.0%) of age. Almost half of the respondents are employed by a company (50.4%). Others are self-employed (21.7%), owners with employees (17.1%), employed by a municipality (3.9%) or other (7.0%). Work regions were spread over the Netherlands and covered all provinces. A majority (72.9%) indicated to be member of one of the two Dutch associations for pest controllers (NVPB and PLA..N.). Almost half (47.3%) of the respondents took a post-graduate (one-day) course in ethics for pest managers. Around two thirds (63.6%) of the respondents have one or more hobby or companion animals, among which companion rodents (hamsters and guinea pigs, but no rats and mice).

### 3.2. Effects of Grouping Variables

No statistically significant differences were found for the grouping variables (factors: ethics course, companion animal, member of an association) for most test variables. Only for some test variables of the topics ‘*solutions for problems in practice*’ and ‘*attitude towards IPM*’, differences were found for membership of an association for pest controllers. These results will be shown in the particular results section.

### 3.3. General Attitude of Liminal Rodents and IPM

**Figure 1:**
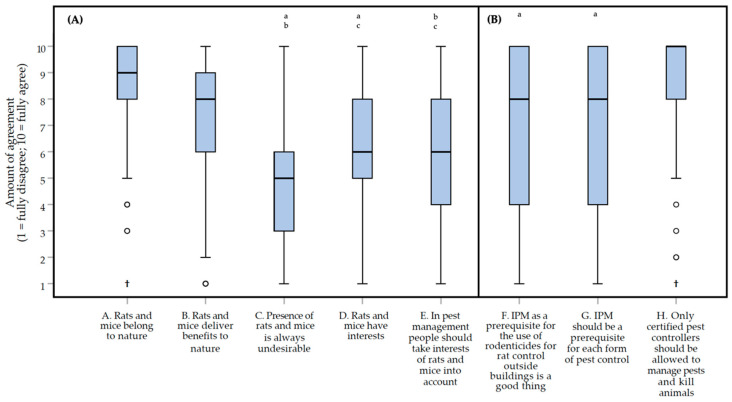
Box plots presenting the amount of agreement with statements about the general attitudes of rats (*Rattus rattus* and *Rattus norvegicus*) and mice (*Mus musculus*) (panel (**A**), statements A–E) and IPM (Integrated Pest Management) regulations (panel (**B**), statements F–H). The amount of agreement could be indicated on a 1 (fully disagree) to 10 (fully agree) interval rating scale. Interests of rats and mice were defined as living, freedom, and welfare. Data were obtained through an online survey among 129 Dutch pest controllers. Outliers and extreme cases are indicated with o and †, respectively. Differences between two statements that ***are not*** statistically significant are indicated with lower case letters above the bars.

The box plots in Figure 1 show the responses to the statements about general attitudes of rats and mice and IPM. There was a significant difference between the scores of the five statements (A–E, Figure 1A) regarding the general attitude of liminal rodents (Friedman repeated measures test, *df* = 4: *Χ^2^* = 192.724, *p* < 0.0000005*, **moderate** effect, *W* = 0.373). The pairwise comparison of the five statements showed that only the scores for statement E (‘*In pest management, people should take interests of rats and mice into account*’) vs. statement C (‘*Presence of rats and mice is always undesirable*’), the scores for statement E vs. those for statement D (‘*Rats and mice have interests*’), and the scores for statement C vs. those for statement D were not significantly different from each other (Wilcoxon matched-pairs signed-ranks test: E vs. C, *Z* = −2.494, *p* = 0.012, *small* effect, |*r*| = 0.155; E vs. D, *Z* = −1.405, *p* = 0.16, zero or nearly zero effect, |*r*| = 0.087; C vs. D, Z = −3.484, *p* = 0.000414, *small* effect, |*r*| = 0.217). Most respondents agreed with statements A that ‘*Rats and mice belong to nature*’ and B that ‘*Rats and mice deliver benefits to nature*’. With a median score of 9 (on the 1 to 10 scale; IQR = 2) and 77.5% of respondents giving a score of 8 or higher for statement A, and a median score of 8 (IQR = 3) and 79.8% of respondents giving a score of 6 or higher for statement B. Respondents tended to disagree with statement C that ‘*The presence of rats and mice is always undesirable*’, with a median score of 5 (IQR = 3.5) and 55.8% of the respondents scoring between 3 and 6. Respondents tended to agree with statement D that ‘*rats and mice have interests*’; with a median score of 6 (IQR = 3) and 64.3% of the respondents scoring between 5 and 8. Respondents scored somewhat neutral for statement E that ‘*In, pest management people need to take the interests of rats and mice into account*’, with a median score of 6 (IQR = 4) and 68.2% of respondents scoring between 4 and 8. Exact *p* values and effect sizes per *post-hoc* comparison, can be found in Supplementary Table S2.

There was a significant difference between the scores of the three statements (F–H, Figure 1B) regarding attitude towards IPM (Friedman repeated measures test, *df* = 2: *Χ^2^* = 67.607, *p* < 0.0000005*, *small* effect, *W* = 0.262). The pairwise *post-hoc* comparisons of the three statements showed that the scores for statement H (‘*Only certified pest controllers should be allowed to manage pests and kill animals*’) vs. those for statement F (‘*IPM as a prerequisite for the use of rodenticides for rat control outside buildings is a good thing*’) or scores for statement H vs. those for statement G (‘*IPM should be a prerequisite for each form of a pest control*’) were significantly different from each other (Wilcoxon matched-pairs signed-ranks test: F vs. G, *Z* = −1.624, *p* = 0.105, *small* effect, |*r*| = 0.101; F vs. H, *Z* = −5.499, *p* < 0.0000005*, **moderate** effect, |*r*| = 0.342; G vs. H, *Z* = −5.883, *p* < 0.0000005*, **moderate** effect, |*r*| = 0.366). Respondents were relatively positive about IPM with a median score of 8 (IQR = 6) for the statements F and G. There was, however, quite some variation, with 82.2% and 76.7% of respondents scoring between 4 and 10 for statements F and G, respectively. Respondents fully agreed with the statement that ‘*Only certified pest controllers should be allowed to manage pests and kill animals*’ (statement H) with a median score of 10 (IQR = 2). The exact *p* values and effect sizes per *post-hoc* comparison can be found in Supplementary Table S3.

**Figure 2:**
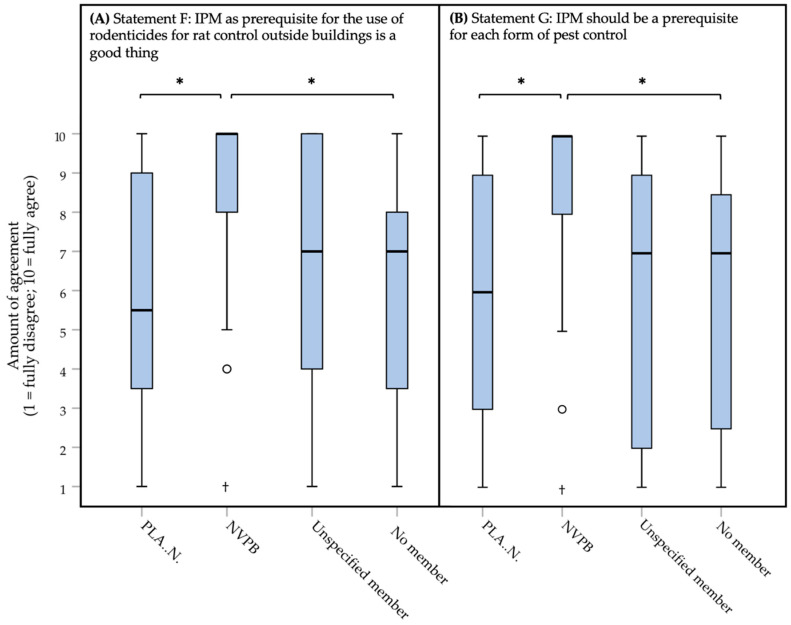
Box plots presenting the amount of agreement with three statements (panel (**A**): statement F; panel (**B**): statement G) about the general attitudes of IPM (Integrated Pest Management). Amount of agreement could be indicated on a 1 (fully disagree) to 10 (fully agree) interval rating scale. Data were obtained through an online survey among the total 129 Dutch pest controllers about the treatment of rats (*Rattus rattus* and *Rattus norvegicus*) and mice (*Mus musculus*). Outliers and extreme cases are indicated with o and †, respectively. Statistically significant differences between two types of membership (PLA..N.: *n* = 40; NVPB: *n* = 35; Unspecified member: *n* = 19; No member: *n* = 35) are indicated with * for each of the panels (**A**,**B**).

When looking deeper into these results, it was found that differences in agreement for these statements exist between different pest controller association memberships. There were significant differences (Kruskal–Wallis test, *df* = 3) between pest controller association memberships for each of the three statements (Figure 2A, statement F: *H* = 22.973, *p* = 0.000100*, *small* effect, *η^2^* = 0.165; Figure 2B, statement G: *H* = 20.642, *p* < 0.0000005*, *small* effect, *η^2^* = 0.147; statement H: *H* = 13.425, *p* = 0.004100*, zero or nearly zero effect, *η^2^* = 0.090). After *post-hoc* testing, it was found that respondents being a member of the NVPB (median of 10, IQR = 2) agreed significantly more with statement F than respondents being a member of PLA..N. (median of 5.5, IQR = 5.75; *U* = 322.5, *W* = 1142.5, *Z* = −4.115, *p* = 0.000023*, **moderate** effect, |*r*| = 0.475) or not being a member (median of 7, IQR = 5; *U* = 250.0, *W* = 880.0, *Z* = −4.369, *p* = 0.000006*, ***large*** effect, |*r*| = 0.522). No significant differences were found between respondents being a member of NVPB and respondents being an unspecified member (median of 7, IQR = 5; *U* = 194.5, *W* = 384.5, *Z* = −2.657, *p* = 0.007, *small* effect, |*r*| = 0.362). Respondents being members of the NVPB (median of 10, IQR = 2) also agreed significantly more with statement G, than respondents being a member of the other association (median of 6, IQR = 6; *U* = 325.5, *W* = 1145.5, *Z* = −4.055, *p* = 0.000031*, **moderate** effect, |*r*| = 0.468) or not being a member (median of 7, IQR = 7; *U* = 289.0, *W* = 919.0, *Z* = −3.886, *p* = 0.000067*, **moderate** effect, |*r*| = 0.464). Also, for this statement, no significant differences were found between respondents being a member of NVPB and unspecified members (median of 7, IQR = 8; *U* = 184.5, *W* = 374.5, *Z* = −2.800, *p* = 0.005, *small* effect, |*r*| = 0.381). After *post-hoc* testing, there were no significant differences (210.0 < *U* < 608.0, 520.0 < *W* < 1339.5, −3.301 < *Z* < −0.062, 0.0008 < *p* < 0.96) between the various categories of pest controller association membership regarding statement H. Exact *p* values and effect sizes per *post-hoc* comparison, can be found in Supplementary Table S10.

### 3.4. Animal Welfare

**Figure 3:**
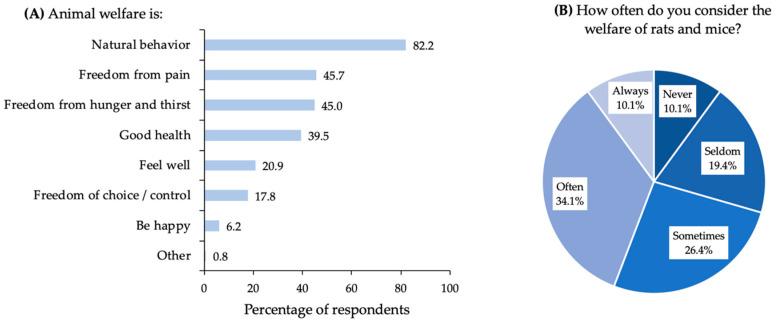
Panel (**A**): bar chart presenting the definition of animal welfare according to Dutch pest controllers. Respondents could choose a maximum of three aspects being important for their understanding of animal welfare. The numbers show the percentage of respondents who chose a certain aspect. In the survey, natural behavior was mentioned as the ‘Possibility to show natural behavior’. Panel (**B**): pie chart presenting how often the welfare of rats (*Rattus rattus* and *Rattus norvegicus*) and mice (*Mus musculus*) is considered by Dutch pest controllers. The results were obtained from 129 Dutch pest controllers who participated in an online survey.

At the beginning of the section about animal welfare, respondents were asked to indicate what their understanding of animal welfare is. Respondents could choose different aspects (maximum of three) they consider being a part of animal welfare. As is shown in Figure 3A, ‘*Natural behavior*’ (in the survey mentioned as ‘possibility to show natural behavior’) was the aspect mostly chosen by 82.2% of respondents, followed by ‘*freedom from pain*’, ‘*freedom from hunger and thirst*’, and ‘*good health*’. A number of 23 respondents (17.8% of the total) chose only a single aspect from the list. ‘*Natural behavior*’ was, in this case, chosen by 20 (87.0%) of these respondents. One respondent chose the option ‘*Other*’, thereby indicating that the definition depends on the location where the animals are. A majority of 60.5% of the respondents indicated to consider the welfare of rats and mice in pest control often or sometimes (see Figure 3B). When respondents were asked to indicate on a 1 (do not want to do more) to 10 (want to do a lot more) scale whether they want to do more for animal welfare in their job, there was quite some variation. Some respondents would like to do (a lot) more, and some do not want to do more at all. Almost half of the respondents (49.6%) gave a score between 4 and 7 with a median of 5 and an IQR of 4.

**Figure 4:**
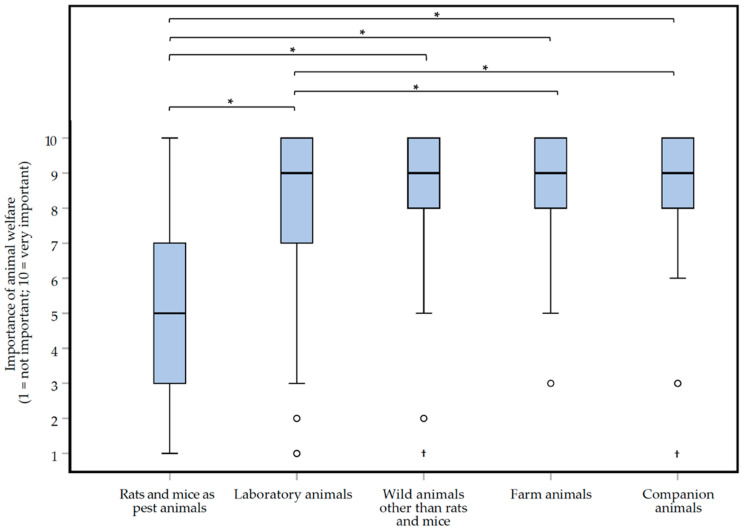
Box plots presenting the scored importance of animal welfare for five different categories of animals according to 129 Dutch pest controllers participating in an online survey about the treatment of rats (*Rattus rattus* and *Rattus norvegicus*) and mice (*Mus musculus*). The importance could be indicated on a 1 (not important) to 10 (very important) interval rating scale. Outliers and extreme cases are indicated with o and †, respectively. Statistically significant differences between two animal categories are indicated with *.

The box plots in Figure 4 show how important respondents found the welfare of rats and mice as pest animals, and relative to the welfare of animals in other contexts. Respondents were somewhat neutral (median score of 5, IQR = 4) about the importance of animal welfare for rodents as pest animals. They found it not unimportant, nor important. Furthermore, there was quite some variation in their answers, with 55.0% of respondents scoring the importance between 4 and 7. This was in contrast with the importance of animal welfare in other categories, for which the interquartile distance was 1.3 to 2 times smaller. Respondents scored the importance of animal welfare differently depending on the animal category (Friedman repeated measures test, *df* = 4: *Χ^2^* = 260.596, *p* < 0.0000005*, ***large*** effect, *W* = 0.505). It was found that respondents scored the importance of welfare for pest rodents (‘*Rats and mice as pest animals*’ median of 5, IQR = 4) significantly (Wilcoxon matched-pairs signed-ranks test) lower (*p* < 0.0000005*) than for the other animal categories; the associated effect sizes were ***large***, |*r*| > 0.5). Furthermore, the importance of welfare for ‘*Laboratory animals*’ was found to be less important than for ‘*Farm animals*’ or ‘*Companion animals*’ (Wilcoxon matched-pairs signed-ranks test: ‘*Laboratory animals*’ vs. ‘*Farm animals*’, *Z* = −4.553, *p* = 0.000001*, *small* effect, |*r*| = 0.283; ‘*Laboratory animals*’ vs. ‘*Companion animals*’, *Z* = −5.887, *p* < 0.0000005*, moderate effect, |*r*| = 0.367). The exact *p* values and effect sizes per *post-hoc* comparison, can be found in Supplementary Table S4.

**Figure 5:**
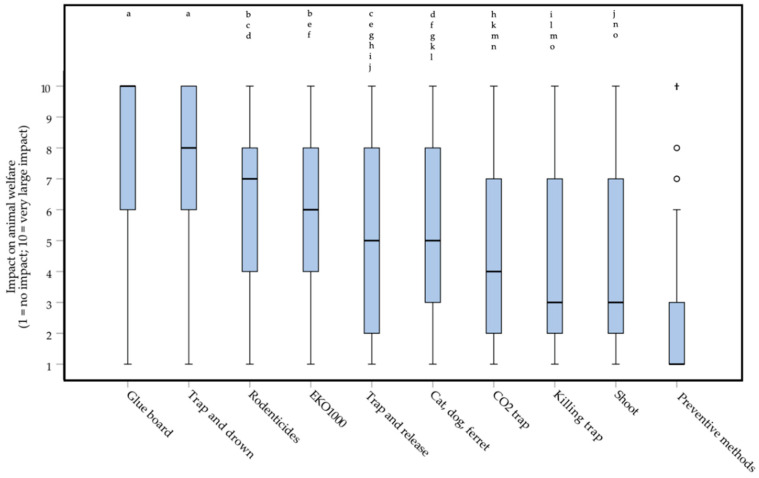
Box plots presenting the welfare impact of ten methods for the control of rats (*Rattus rattus* and *Rattus norvegicus*) and mice (*Mus musculus*), according to 129 Dutch pest controllers participating in an online survey. The impact could be indicated on a 1 (no impact) to 10 (very large impact) interval rating scale. Outliers and extreme cases are indicated with o and †, respectively. Differences between the two methods that ***are not*** statistically significant are indicated with letters above the bars.

Respondents saw differences in the welfare impact of available control methods (Friedman repeated measures test, *df* = 9: *Χ^2^* = 407.691, *p* < 0.0000005^*^, **moderate** effect, *W* = 0.351) (Figure 5). *Post-hoc* testing (Wilcoxon matched-pairs signed-ranks test) showed that they think ‘*Glue boards*’ (median of 10, IQR = 4; maximal impact) and ‘*Trap and drown*’ (median of 8, IQR = 4) have a very high impact on welfare (involving severe suffering), whereas ‘*Killing trap*’, ‘*Shooting*’, and ‘*Preventive methods*’ had a much lower or almost no impact on welfare. The impact scores of ‘*Glue boards*’ and ‘*Trap and drown*’ were rated significantly higher than all other methods (−9.130 < *Z* < −4.786, *p* < 0.0000005*, *small* to ***large*** effects, 0.297 < |*r*| < 0.569), but did not differ significantly from each other (*Z* = −1.636, *p* = 0.10, *small* effect, |*r*| = 0.102). The impact score of ‘*Preventive methods*’ was rated significantly lower than all other methods (−9.130 < *Z* < −5.568, *p* < 0.0000005*, **moderate** to ***large*** effects, 0.346 < |*r*| < 0.569). The methods ‘*Rodenticides*’ and ‘*EKO1000′* scored a significantly higher impact than the methods ‘*CO_2_ trap*’, ‘*Killing trap*’, and ‘*Shooting*’ (−6.671 < *Z* < −4.830, *p* < 0.0000005*, **moderate** effects, 0.301 < |*r*| < 0.416). The method ‘*Cat, dog, ferret*’ scored a significantly higher impact than ‘*Shooting*’ (*Z* = −4.915, *p* < 0.0000005*, **moderate** effect, |*r*| = 0.306).

The control methods used by most respondents were ‘*Killing trap*’ (96.1% of respondents), ‘*Rodenticides*’ (88.4%) and ‘*Preventive methods*’ (87.6%). ‘*Trap and release*’ was used by 47.2% percent of respondents, ‘*EKO1000′* by 35.7%, ‘*CO_2_ trap*’ by 18.6%, ‘*Shooting*’ by 17.1% and ‘*Glue board*’ by 10.9%. Seven percent of respondents indicated using other methods than the ones mentioned in the list.

While ‘*Rodenticides*’ were used by almost 90% of respondents, this method was given a relatively high welfare impact score (median of 7, IQR = 4). Its impact was scored significantly higher (−8.625 < *Z* < −4.786, *p* < 0.0000005*, *small* to ***large*** effects, 0.298 < |*r*| < 0.537) than some commonly used other methods (Figure 5), except when compared to ‘*Trap and release*’ (*Z* = −2.555, *p* = 0.010, *small* effect, |*r*| = 0.159), ‘*Cat, dog, ferret*’ (*Z* = −2.938, *p* = 0.003, *small* effect, |*r*| = 0.183) or ‘*EKO1000′* (*Z* = −1.230, *p* = 0.220, zero or nearly zero effect, |*r*| = 0.077). The exact *p* values and effect sizes per *post-hoc* test, can be found in Supplementary Table S5.

### 3.5. Decisions in Daily Practice

**Figure 6:**
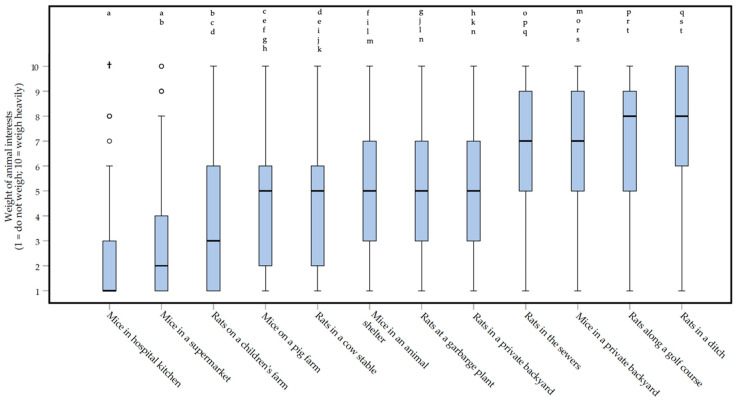
Box plots presenting the importance of the interests of rats (*Rattus rattus* and *Rattus norvegicus*) and mice (*Mus musculus*) in twelve different real-life scenarios in rodent control according to 129 Dutch pest controllers, participating in an online survey. Interests of rats and mice were defined as living, freedom, and welfare. Weight of interests could be indicated on a 1 (do not weigh) to 10 (weigh heavily) interval rating scale. Outliers and extreme cases are indicated with o and †, respectively. Differences between the two scenarios that ***are not*** statistically significant are indicated with letters above the bars.

Besides the scoring of control methods on their welfare impact, respondents were asked to indicate how important animal interests were for different real-life situations in rodent control. Respondents indicated that in different situations, a different weight could be attributed to animal interests (Friedman repeated measures test, *df* = 11: *Χ^2^* = 636.188, *p* < 0.0000005*, **moderate** effect, *W* = 0.448) (Figure 6). *Post-hoc* testing (Wilcoxon matched-pairs signed-ranks test) showed that whereas animal interests were deemed of no or trivial importance in the management/control of ‘*Mice in a hospital setting*’ (median of 1, IQR = 2), animal interests mattered much more when dealing with ‘*Mice in a private backyard*’ (median of 7, IQR = 4.5) or ‘*Rats in a ditch*’ (median of 8, IQR = 4). For ‘*Mice in a hospital kitchen*’, the weight of animal interests was scored significantly lower (−9.117 < *Z* < −5.874, *p* < 0.0000005*, **moderate** to ***large*** effects, 0.366 < |*r*| < 0.568) than those for all other scenarios, except for ‘*Mice in a supermarket*’ (*Z* = −3.829, *p* = 0.000072, *small* effect, |*r*| = 0.238). The score for ‘*Rats in a ditch*’ was significantly higher (−9.117 < *Z* < −7.405, *p* < 0.0000005*, **moderate** to ***large*** effects, 0.460 < |*r*| < 0.568) than for all other scenarios, except when compared to ‘*Rats along a golf court*’ (*Z* = −2.288, *p* = 0.02, *small* effect, |*r*| = 0.142), ‘*Rats in the sewers*’ (*Z* = −3.660, *p* = 0.0002, *small* effect, |*r*| = 0.228), and ‘*Mice in a private backyard*’ (*Z* = −2.288, *p* = 0.00003, *small* effect, |*r*| = 0.252). The score for ‘*Mice in a supermarket*’ was significantly higher (−8.749 < *Z* < −5.528, *p* < 0.0000005*, **moderate** to ***large*** effects, 0.366 < |*r*| < 0.568) than for all other scenarios, except for ‘*Mice in a hospital kitchen*’ (*Z* = −3.829, *p* = 0.00007, *small* effect, |*r*| = 0.238) and ‘*Rats on a children*’*s farm*’ (*Z* = −2.399, *p* = 0.02, *small* effect, |*r*| = 0.149). Scores with a median of 5, were given to five of the scenario’s *(*‘*Mice on a pig farm*’, ‘*Rats in a cow stable*’, ‘*Mice in an animal shelter*’, ‘*Rats at a garbage plant*’, and ‘*Rats in a private backyard*’; Figure 5). There were no significant differences between these scenarios (−3.800 < *Z* < −0.161, 0.00009 < *p* < 0.874, zero or nearly zero to *small* effects, 0.010 < |*r*| < 0.237). In Supplementary Table S6, the exact *p* values and effect sizes can be found. Besides the differences between scenarios, the results indicated that respondents saw differences between rats and mice. For example, the weight of interests of ‘*Mice in a private backyard*’ was scored significantly higher than the interests of ‘*Rats in a private backyard*’ (*Z* = −6.804, *p* < 0.0000005*, **moderate** effect, |*r*| = 0.424).

### 3.6. Problems and Solutions in Daily Practice

In the survey, almost half of the respondents (45.7%) indicated encountering problems when weighing rodent interests (e.g., animal welfare, being alive) against human interests (e.g., costs, food safety, hygiene). Fourteen percent of respondents didn’t know and the rest (40.3%) indicated to not encounter problems. The majority (64.9%) of the problems indicated by respondents (open response question) were client-related. According to the respondents, a lot of clients lack the willingness to invest sufficient money or efforts in preventive methods, the latter being a relatively animal-friendly control method in the eyes of respondents (Figure 5). Furthermore, they think prevention is an effective control method. When they were asked to indicate what percentage of nuisance could be taken away by using preventive methods only, the average was 62.6%, with a standard deviation of 23.2%.

**Figure 7:**
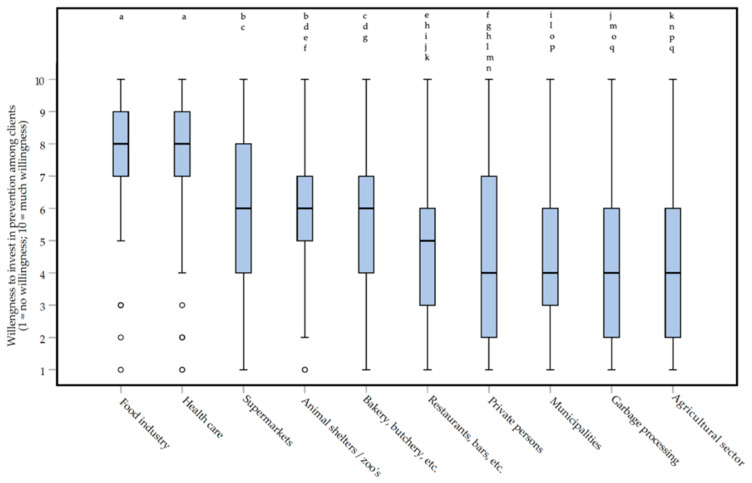
Box plots presenting the willingness to invest in prevention among clients in different sectors on a 1 (no willingness) to 10 (much willingness) rating scale. Willingness was indicated by 129 Dutch pest controllers, participating in an online survey about the treatment of rats (*Rattus rattus* and *Rattus norvegicus*) and mice (*Mus musculus*). Differences between the two clients that ***are not*** statistically significant are indicated with letters above the bars.

The extent to which clients are willing to invest in preventive methods differs across sectors (e.g., food production, agricultural, health care, governments) where clients belong to (Friedman repeated measures test, *df* = 9: *Χ^2^* = 242.965, *p* < 0.0000005*, **moderate** effect, *W* = 0.342) (Figure 7). *Post-hoc* testing (Wilcoxon matched-pairs signed-ranks test) showed that respondents think that clients in food industry and health care (medians of 8 and IQRs of 2) invest significantly more than clients in other sectors (−8.703 < Z < −5.339, *p* < 0.0000005*, **moderate** to ***large*** effects, 0.331 < |*r*| < 0.542). Clients in the agricultural sector, garbage processing, and municipalities invest significantly lower than clients in the food industry, health care, animal shelters or zoo’s, supermarkets, and bakery or butchery, etc. (−8.216 < *Z* < −4.693, *p* < 0.0000005*, *small* to ***large*** effects, 0.292 < |*r*| < 0.512), according to respondents. See Supplementary Table S7 for all the exact *p*-values and effect sizes.

**Figure 8:**
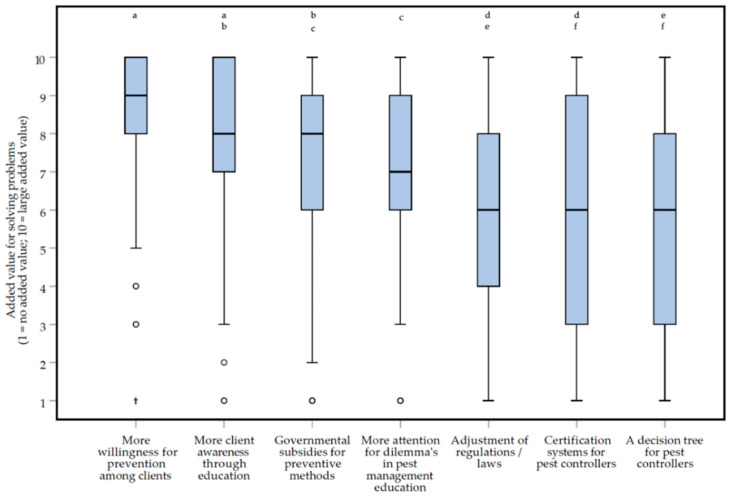
Box plots presenting the added value of different possible solutions for problems in practice on a 1 (no added value) to 10 (large added value) rating scale. Solutions were scored by 129 Dutch pest controllers, participating in an online survey about the treatment of rats (*Rattus rattus* and *Rattus norvegicus*) and mice (*Mus musculus*). Differences between the two solutions that ***are not*** statistically significant are indicated with letters above the bars.

Respondents think that more client awareness and willingness to invest in prevention are the best solutions to overcome the problems faced in practice (Figure 8). After initial testing (Friedman repeated measures test, *df =* 6: *Χ^2^* = 219.467, *p* < 0.0000005*, *small* effect, *W* = 0.284), *post-hoc* testing (Wilcoxon matched-pairs signed-ranks test) showed that clients awareness (median of 8, IQR = 3) and willingness (median of 9, IQR = 2) to invest in prevention as solutions were given a high score by respondents and were scored significantly higher than almost all other possible solutions provided (−8.384 < *Z* < −4.850, *p* < 0.0000005*, **moderate** to ***large*** effects, 0.301 < |*r*| < 0.522), except ‘*Governmental subsidies for preventive methods*’ (Z = −2.954, *p* = 0.003, *small* effect, |*r*| = 0.184). In Supplementary Table S8, the exact *p* values and effect sizes can be found.

**Figure 9:**
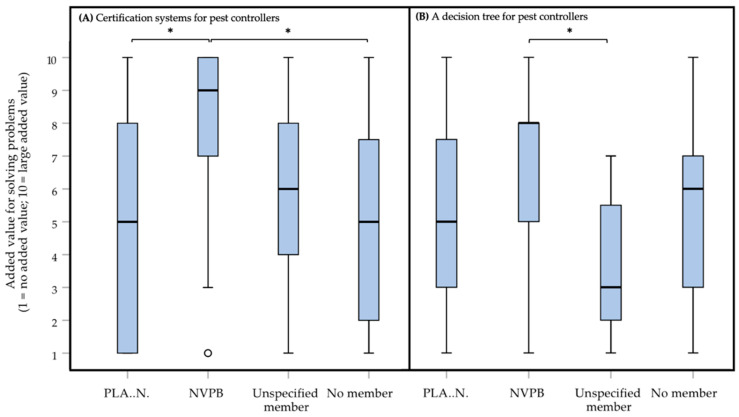
Box plots presenting the added value of different possible solutions (panel (**A**): certification systems for pest controllers; panel (**B**): a decision tree for pest controllers) for problems in practice on a 1 (no added value) to 10 (large added value) rating scale. Solutions were scored by in total 129 Dutch pest controllers, participating in an online survey about the treatment of rats (*Rattus rattus* and *Rattus norvegicus*) and mice (*Mus musculus*). Outliers are indicated with o. Statistically significant differences between the two types of membership (PLA..N.: *n* = 40; NVPB: *n* = 35; Unspecified member: *n* = 19; No member: *n* = 35) are indicated with * for each of the panels (**A**,**B**).

When looking deeper into the scores for added value of possible solutions it was found that differences (Kruskal–Wallis test, *df* = 3) in scores exist between different pest controller association memberships. Differences were found for the scores for ‘*Adjustment of regulations/laws*’ (*H* = 15.608, *p* = 0.001100*, *small* effect, *η^2^* = 0.101), ‘*Certification systems for pest controllers*’ (*H* = 20.369, *p* < 0.0000005*, *small* effect, *η^2^* = 0.139; Figure 9A) and ‘*A decision tree for pest controllers*’ (*H* = 20.369, *p* < 0.0000005*, *small* effect, *η^2^* = 0.139; Figure 9B). In *post-hoc* testing (Wilcoxon–Mann–Whitney test) the differences between the pest controller association memberships for the scores for ‘*Adjustment of regulations/laws*’ did not reach the level of statistical significance (187.0 < *U* < 684.5, 377.0 < *W* < 1314.5, −3.338 < *Z* < −0.166, 0.0006 < *p* < 0.87). Respondents being a member of NVPB (median of 9, IQR = 3) were more positive about ‘*Certification systems for pest controllers*’ than respondents being a member of PLA..N. (median of 5, IQR = 7; *U* = 326.0, *W* = 1146.0, *Z* = −4.014, *p* = 0.000037*, **moderate** effect, |*r*| = 0.464) or respondents not being a member of an association (median of 5, IQR = 6; *U* = 311.0, *W* = 941.0, *Z* = −3.579, *p* = 0.000257*, **moderate** effect, |*r*| = 0.428). Finally, respondents being a member of NVPB (median of 8, IQR = 3) were more positive about ‘*A decision tree for pest controllers*’ than respondents being an unspecified member (median of 3, IQR = 4; *U* = 103.0, *W* = 292.0, *Z* = −4.190, *p* = 0.000009*, ***large*** effect, |*r*| = 0.501). The exact *p* values and effect sizes per *post-hoc* comparison, can be found in Supplementary Table S10.

**Figure 10:**
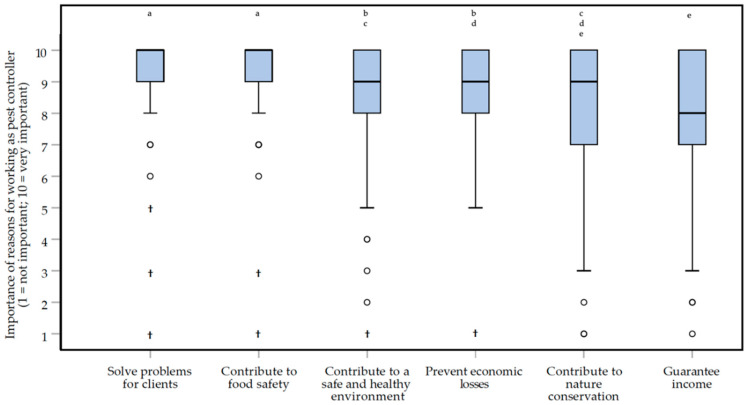
Box plots presenting the importance of the reasons to work as a pest controller on a 1 (not important) to 10 (very important) rating scale. Reasons to work as a pest controller were scored by 129 Dutch pest controllers, participating in an online survey about the treatment of rats (*Rattus rattus* and *Rattus norvegicus*) and mice (*Mus musculus*). Differences between the two reasons that ***are not*** statistically significant are indicated with letters above the bars.

According to respondents, it is important that clients know the real cause of nuisance they experience and should be prepared to invest more in prevention (e.g., cleaning, storing food items, closing holes in buildings). There was a significant difference between the scores of the work motivation of the respondents (Friedman repeated measures test, *df =* 5: *Χ^2^* = 123.312, *p* < 0.0000005*, *small* effect, *W* = 0.191) (Figure 10). Respondents indicated that their main work motivation was to ‘*solve problems for clients*’, together with ‘*contributing to food safety*’ (median scores of 9 and an IQR of 1 and 1.5, respectively). These two reasons to work as a pest controller were scored significantly higher than other reasons provided (Wilcoxon matched-pairs signed-ranks test; 8.384 < *Z* < −4.850, *p* < 0.000043*, *small* to **moderate** effects, 0.245 < |*r*| < 0.408). Exact *p* values and effect sizes for work motivation can be found in Supplementary Table S9.

## 4. Discussion

Attention to moral status and animal welfare in the treatment of liminal rodents perceived as pests seems scarce. At the same time, stakeholders consider this a pressing issue [14]. The present study is part of an ongoing project to shed light on this area. It does so in the spirit of a bottom-up approach, starting at the grassroots level to uncover relevant considerations and moral issues, generating engagement in the process. For CenSAS, taking the perspective of the animal is key. The shared commitment among stakeholders to take the interests of animals seriously in pest management strategies provides an important condition to participate and facilitate this process. Beyond promoting collaboration between stakeholders and managing the project, CenSAS contributes by means of expertise regarding animals and their interests, including both animal welfare science and philosophy (ethics in particular). The goal of the project is to develop an assessment frame for responsible pest management, which can be used by the professionals in practice. In light of the bottom-up approach, the input of pest controllers is indispensable. Therefore, an online survey among Dutch pest controllers was carried out to learn more about their attitudes regarding animal welfare in rodent control, their experiences of (moral) dilemmas in practice, and possible ways (among which the use of an assessment frame) to overcome these dilemmas.

The respondents of the survey believe that rats and mice have interests (e.g., living, freedom, welfare), and they are, in general, quite positive about IPM and the effects preventive methods can have (Figure 1). These findings are relevant if one wants to develop an assessment frame that starts with prevention, and which has to weigh potential harm to the interests of animals against the interests of humans. The results show that respondents being a member of the NVPB were more positive about IPM than other respondents, except for unspecified members (Figure 2). At the moment, we are unable to fully explain this finding. They may be more positive about regulations and guidance for good practices in general, since these respondents also tended to be more positive about ‘*Adjustments of regulations*’, ‘*Certification systems*’, and ‘*A decision tree*’ as solutions for overcoming problems in practice. All respondents agreed with the statement that only certified pest controllers should be allowed to manage pests. This may indicate that they view their job as a profession that should be acknowledged as such.

The results show that the respondents do consider animal welfare during their job (Figure 3B). The differences found in the scoring of the importance of animal welfare (Figure 4), supports earlier findings of a variety or even inconsistencies in the treatment of animals, depending on the context [18]. What precisely causes these differences to arise remains somewhat unclear. Likely various aspects, such as a difference in the (moral) evaluation of liminal rodents in comparison to other animals [1], the plurality of views on the moral status of animals [19], the difference due to ethical choices in the weighing between animal welfare and human interests, and the importance of moral intuitions [20], including the ‘yuck factor’ [21] play a role.

Surprisingly, no statistically significant differences were found for the grouping variable ‘ethics course’. This was unexpected, since one may assume to find that respondents who took a post-graduate ethics course (with a focus on animal welfare and weighing animal against human interests) would have a different attitude towards the treatment and welfare of rats and mice, especially when it comes to the weighing of animal interests against human interests. At the moment, we are unable to explain this finding. Focus group dialogues should shed more light on both this finding and the differences found in the importance of welfare depending on the animal category.

The great majority of respondents indicated using rodenticides. At the same time, they score this method as one with a high impact on welfare (Figure 5). This finding may indicate the existence of a ‘rodenticide dilemma’ and may point at a need for new control methods, which are both effective and less harmful to the animals involved. It might be useful to discuss and investigate this further to see if pest controllers do indeed experience this as a dilemma or not. Also, it should be discussed if methods with the highest welfare impact (glue trap, drowning) should be used at all. The welfare impact scores are given by pest controllers. It tells us how they think about different methods, but it does not tell the real welfare impact. Scoring based on scientific knowledge and expertise in this particular field is necessary to rate the impact of methods in an objective way. The model developed by Sharp and Saunders [22] could be a useful tool to do so. This model constitutes two parts, one part looks at the impact of control methods based on overall welfare and the duration of the impact. The other part looks at the effects of a killing method by looking at the intensity and duration of suffering caused. With this model, the welfare impact can be analyzed thoroughly.

Animal welfare comprises an important building block for the assessment framework. Just as various conceptions of animal welfare exist in the literature, e.g., [23,24], a broad and diverse range also existed among stakeholders [14]. How should we deal with a plurality of views regarding the meaning of such a crucial concept? We have distinguished between suffering as a measure of animal welfare to assess the impact of pest management methodologies, and animal interests as an open-ended value for the participant to consider in brief examples of cases. In addition, we ask the participants to indicate what best approximates animal welfare out of the following features—the absence of suffering, health, natural behavior, feeling good, and control regarding one’s life (Figure 3A). Natural behavior turned out to be the most important aspect of welfare for the respondents. Animal welfare as a concept in the management of liminal rodents will be explored further elsewhere as part of the ongoing project.

As for the welfare impact scores of control methods, the scores for weighing animal interests show some grey areas (Figure 6). Together, these findings offer the potential for in-depth discussions within the focus groups. Furthermore, the combination of welfare impact scores and animal interest scores could be used to develop an assessment frame to facilitate responsible rodent management. On the one hand, these findings suggest there may be practical situations in which the animal interests are deemed less important, even to the extent of considering more harmful control methods. On the other hand, there are situations in which animal interests are explicitly taken into account, and only less harmful or even no control methods are deemed acceptable. Besides the differences between scenarios, respondents see differences between rats and mice. Whether one frame for both species will do, therefore, remains unclear. Maybe there should be separate frames for different species, or perhaps the frame should question the moral relevance of species.

Respondents indicated encountering problems in practice and they attributed these problems to clients. At the same time, they deemed client satisfaction important for work motivation (Figure 10). The combination of these findings may point at another dilemma. On the one hand, the pest controllers want to assure client satisfaction, while on the other hand, they sometimes need to be critical of client behaviors and attitudes. This calls for strong communicative skills as indispensable for pest management professionals, both in terms of fostering ecological as well as moral awareness in clients. In order to promote investment in preventive methods, pest management professionals have to be able to educate clients on the ecological drivers of the existing conflict with liminal rodents. In moral terms, pest management professionals have to engage clients in ethical decision making and diminish strong biases in this process [25]. It is worth exploring whether the assessment-frame as a communication tool can facilitate pest management in these capacities and support them in the dilemmas they face.

The initial idea of the ongoing project was to develop an assessment frame (in the survey the Dutch word ‘*Beslisboom*’, meaning decision tree, was used) for pest controllers specifically. Noteworthy, a decision tree for pest controllers as a possible solution for problems in practice (Figure 8), together with the adjustment of regulations and certification systems for pest controllers, scored the lowest (medians of 6), and scored significantly lower than the other solutions provided (*p*-values < 0.0000005, and absolute effect sizes |*r*|, ranging from 0.1 to 0.5). Taking this into account, in addition to the findings of client awareness, an assessment frame that could be used by both pest controllers and their clients together may be more helpful to overcome the identified problems. Besides ethical decision-making, such a frame may function as a communication tool and improve the controller-client relationship. We will look into this further in focus groups and the continuation of our research. Variation between pest controllers may exist, since respondents being a member of NVPB were more positive about ‘*A decision tree for pest controllers*’ as a solution for problems in practice than respondents being an unspecified member (Figure 9).

One perhaps wonders how the assessment frame relates to earlier suggestions to extrapolate ethical assessment frameworks from animal research ethics to the context of pest management [6]. First of all, (1) our objective is to integrate concern for animals at the level of practice, bottom-up, whereas these ethical assessment methods indicated in the literature—which could prove valuable in managing conflicts between humans and liminal rodents—are extrapolated from another field, to be introduced top-down. Authorities could for example follow up on the suggestions made in the literature and require that all pest management measures require the application of the three Rs and a harm-benefit analysis. We have seen, however, that these kinds of solutions may not prove very beneficial in the eyes of the respondents, and there are further methodological questions to be addressed as well [26]. Our bottom-up approach starts off from the concerns raised by professionals themselves. Taken together with the shared commitment among stakeholders to work towards responsible pest management, our approach not only generates a support base but also promotes moral awareness by inviting professionals to reflect on the questions raised in the survey. As such, the resulting assessment frame on this approach will reflect and build upon moral engagement within the profession itself.

As a second point (2), we need to establish whether these tools are adequate in themselves. To what extent should we take these methods at face value, ready for extrapolation? What exactly happens when animal research is justified by means of a harm-benefit analysis? To what extent does one’s perception (including emotional engagement) of the animals in question introduce bias, either explicitly or implicitly, in ethical assessment? We explore these questions more in-depth elsewhere [25].

As a third point (3), if prevention becomes an important part of management strategies, the interaction between humans and liminal rodents appears to move more towards co-existence rather than mere conflict. Such a shift in perspective will likely require new sources, novel “tools” to think about living together with animals. This could prove a next step in the development from “pest eradication” to “pest management”, perhaps resulting in consultancy, primarily aimed at mediating the interests of both humans and liminal rodents [25].

As a final point (4), we are interested in the professional agency of individual pest management professionals. This is why the current survey, as we hypothesize, needs to be deepened by focus groups, as well as put into the context of determinants that shape the range of professional agency of pest management professionals. How does professional training shape their capabilities to negotiate the demands of clients, employers, society, and the liminal rodents [27]? To what extent should we expect change to arise from individual professionals, supported by an assessment frame, rather than as a result of more general regulations and restrictions? In order to develop frameworks that truly support the decision-making of individual professionals, unravelling the underlying drivers that shape current pest management strategies emerges as the anticipated next step.

## 5. Conclusions

Dutch pest controllers participating in the present survey indicate that they take the welfare of pest rodents into consideration as part of their job. They do, however, deem the welfare of pest rodents less important than the welfare of other animals. Existing control methods are scored differently in terms of their welfare impact. Rodenticides are used by a great majority of the respondents but are given quite a high welfare impact score. We refer to this as the ‘rodenticide dilemma’. This may point to a need for other control methods, which are both effective and less harmful. The weight attributed to the interests (e.g., living, freedom and welfare) of liminal rodents depends on the type of real-life situation (mice in a hospital vs. rats in a ditch). These findings will form the assessment frame for more responsible rodent management. Almost half of the respondents encounter problems or difficulties when they need to weigh the interests of rodents against those of humans. Most of these problems are related to the respondents to clients who do not want to invest enough in preventive methods. Respondents point at the need for more client awareness and motivation for prevention. For this reason, an assessment frame that can be used by both pest controllers and clients together, appears more beneficial and can serve as a communication tool to support the pest controller-client relationship. No statistically significant differences were found for the grouping variable ‘ethics course’ or ‘companion animal’. Statistically significant differences were found for the variable ‘membership of an association for pest controllers. Members of NVPB seemed to be more positive about IPM and were more positive about the certification systems, and a decision tree as solutions for overcoming problems in practice.

The results of the survey form a relevant basis for broader discussions about the treatment of liminal rodents with, for example, clients of pest controllers and in-depth conversations with pest controllers in focus groups. Outcomes of the survey and discussions will be used in the development of the assessment frame for responsible and sustainable liminal rodent management.

## Figures and Tables

**Table 1:** Thresholds for interpreting effect size.

	Thresholds Used for Qualitative Descriptions of Effect Size ^2^			
Effect Size Indices ^1^	Zero or Nearly Zero Effect	*Small* Effect	**Moderate** Effect	***Large*** Effect
*W*	0 < *W* ≤ 0.1	0.1 < *W* ≤ 0.3	0.3 < *W* ≤ 0.5	*W* > 0.5
*η* ^2^	0 < *η*^2^ ≤ 0.01	0.01 < *η*^2^ ≤ 0.06	0.06 < *η*^2^ ≤ 0.14	*η*^2^ > 0.14
*r*	0 < |*r*| ≤ 0.1	0.1 < |*r*| ≤ 0.3	0.3 < |*r*| ≤ 0.5	|*r*| > 0.5

^1^ Kendall’s *W* values were generated by IBM^®^ SPSS^®^ Statistics, whereas *η^2^* and *r* values were computed via an online calculator [16]). ^2^ There is no consensus of to what constitutes a zero or nearly zero, *small*, **moderate** or ***large*** effect size. Cohen [17] gave some rules of thumb for the thresholds.

## Supplementary Materials

The following are available online at https://www.mdpi.com/2076-2615/10/9/1614/s1, Table S1: Overview of the corrected thresholds for statistical significance, Table S2: Exact *p* values and effect sizes for general attitudes about liminal rodents, Table S3: The exact *p* values and effect sizes for general attitudes about IPM (Integrated Pest Management), Table S4: The exact *p* values and effect sizes for the importance of animal welfare between animal categories, Table S5: The exact *p* values and effect sizes for welfare impact of control methods, S6: The exact *p* values and effect sizes for the weight of animal interests for different scenarios, S7: The exact *p* values and effect sizes for client investments in prevention, S8: The exact *p* values and effect sizes for solutions for problems in practice, S9: Exact *p* values and effect sizes for work motivation, S10: Exact *p* values and effect sizes of independent samples.

## Abbreviations

CenSAS	Centre for Sustainable Animal Stewardship
IPM	Integrated Pest Management
IQR	Interquartile range
NGOs	Non-governmental organizations
NVPB	Nederlandse Vereniging Plaagdiermanagement Bedrijven
PLA..N.	Platform Plaagdierbeheersing Nederland
