# Supplementary material for: Dilemmas in the Management of Liminal Rodents—Attitudes of Dutch Pest Controllers
Source: Animals (Basel). 2020 Sep 9;10(9):1614. doi: 10.3390/ani10091614 (PMC7552245; doi:10.3390/ani10091614)
Supplement: Supplementary file 1 [file animals-10-01614-s001.zip › animals-918590-supplementary.pdf]

Supplementary tables to: Dilemmas in the Management of Liminal Rodents – Attitudes of Dutch Pest Controllers

Table S1: Overview of the corrected thresholds for statistical significance

			Wilcoxon-Mann-Whitney test	
	Omnibus tests	*Post hoc* tests	*Omnibus* tests	*Post hoc* tests
Topic<br>● *number of test variables*	Friedman repeated measures test *or* Kruskall-Wallis test	Wilcoxon matched-pairs signed ranks test	Ethics course (Yes/No) *or* Companion (Yes/No) animal (Yes/No)	Member of association (NVPB/PLA..N./Yes, unspecified/No)
	$\gamma = t \times o$[1]	$\gamma = t \times o \times c$	$\gamma = t \times o$	$\gamma = t \times o \times c$
General attitude of liminal rodents<br>● *5 test variables*				
γ	5 x 4 = 20	5 x 4 x 10 = 200	5 x 4 = 20	5 x 4 x 6 = 120
α_{adj}[2]	0.002561	0.000256	0.002561	0.000427
General attitude of IPM<br>● *3 test variables*				
γ	3 x 4 = 12	3 x 4 x 3 = 36	3 x 4 = 12	3 x 4 x 6 = 72
α_{adj}	0.004263	0.001423	0.004263	0.000712
Animal welfare in context<br>● *5 test variables*				
γ	5 x 4 = 20	5 x 4 x 10 = 200	5 x 4 = 20	5 x 4 x 6 = 120
α_{adj}	0.002561	0.000256	0.002561	0.000427
Want to do more for animal welfare<br>● *1 test variable*				
γ	1 x 3 = 3	not applicable	1 x 3 = 3	1 x 3 x 6 = 18
α_{adj}	0.016952	not applicable	0.016952	0.002846
Welfare impact of methods<br>● *10 test variables*				
γ	10 x 4 = 40	10 x 4 x 45 = 1800	10 x 4 = 40	10 x 4 x 6 = 240
α_{adj}	0.001282	0.000028	0.001282	0.000214
Weight of animal interest in different real-life scenarios<br>● *12 test variables*				
γ	12 x 4 = 48	12 x 4 x 665 = 3168	12 x 4 = 48	12 x 4 x 6 = 288
α_{adj}	0.001068	0.000016	0.001068	0.000178
Solutions for problems in practice<br>● *7 test variables*				
γ	7 x 4 = 28	7 x 4 x 21 = 588	7 x 4 = 28	7 x 4 x 6 = 168
α_{adj}	0.001830	0.000087	0.001830	0.000305
Work motivation<br>● *6 test variables*				
γ	6 x 4 = 24	6 x 4 x 15 = 360	6 x 4 = 24	6 x 4 x 6 = 144
α_{adj}	0.002135	0.000142	0.002135	0.000356
Willingness for prevention<br>● *6 test variables*				
γ	6 x 4 = 24	6 x 4 x 15 = 360	6 x 4 = 24	6 x 4 x 6 = 144
α_{adj}	0.002135	0.000142	0.002135	0.000356

1 γ is the number of hypotheses tested (*omnibus* tests: 'number of test variables' (t) multiplied by 'the number of *omnibus* tests performed' (o); *post hoc* tests: 'number of test variables' (t) multiplied by 'the number of *omnibus* tests performed'(o) multiplied by 'the number of pair wise comparisons'(c) per test variable)

2 The formula for calculating the *adjusted alpha* is: $\alpha_{adj} = 1 - [1-\alpha]^{1/\gamma}$, where $\alpha = 0.05$

Table S2: Exact *P* values and effect sizes for general attitudes about liminal rodents

	**A. RM belong to nature (9;10-8)**	**B. RM deliver benefits to nature (8;9-6)**	**C. Presence of RM is always undesirable (5;6-3)**	**D. RM have interests (6;8-5)**	**E. People should take interests of RM into account (6;8-4)**
**A. RM belong to nature (9;10-8)**		0.4078	0.4532	0.5170	0.5309
**B. RM deliver benefits to nature (8;9-6)**	<0.0000005*		0.3463	0.3412	0.3745
**C. Presence of RM is always undesirable (5;6-3)**	<0.0000005*	<0.0000005*		0.2169	0.1553
**D. RM have interests (6;8-5)**	<0.0000005*	<0.0000005*	0.000414		0.0875
**E. People should take interests of RM into account (6;8-4)**	<0.0000005*	<0.0000005*	0.01226	0.161569	

Table S2: Exact (2-tailed) *P* values (under de diagonal line) and absolute effect sizes (above the diagonal line) for differences between statements about general attitudes towards rats (*Rattus rattus* and *Rattus norvegicus*) and mice (*Mus musculus*). Exact (2-tailed) *P* values were calculated for each pair of methods with the Wilcoxon matched-pairs signed ranks test for 2 dependent variables. Numbers in parentheses display the median and the interquartile range (Q3-Q1). Significant *P* values are marked with an asterisk (*). Effect sizes (r) were calculated using the formula $r = z / \sqrt{n}$, where 'n' is the number of observations, in this case 2 x 129 = 258. Thresholds used for qualitative descriptions of effect size were: zero or nearly zero effect, $0 < |r| \leq 0.1$; small, $0.1 < |r| \leq 0.3$; moderate, $0.3 < |r| \leq 0.5$; large $0.5 < |r| \leq 0.7$; and very large, $|r| > 0.7$. Data were obtained through an online survey among 129 Dutch pest controllers. Importance could be indicated on a 1 (not important) to 10 (very important) interval rating scale.

Table S3: Exact *P* values and effect sizes for general attitudes about IPM

	**F. IPM for rat control is a good thing (8;10-4)**	**G. IPM should be a prerequisite for each form of control (8;10-4)**	**H. Only certified pest controllers should be allowed to manage pest (10;10-8)**
**F. IPM for rat control is a good thing (8;10-4)**		0.1011	0.3424
**G. IPM should be a prerequisite for each form of control (8;10-4)**	0.105266		0.3663
**H. Only certified pest controllers should be allowed to manage pest (10;10-8)**	<0.0000005*	<0.0000005*	

Table S3: Exact (2-tailed) *P* values (under de diagonal line) and absolute effect sizes (above the diagonal line) for differences between statements about general attitudes towards IPM (Integrated Pest Management). Exact (2-tailed) *P* values were calculated for each pair of methods with the Wilcoxon matched-pairs signed ranks test for 2 dependent variables. Numbers in parentheses display the median and the interquartile range (Q3-Q1). Significant *P* values are marked with an asterisk (*). Effect sizes (r) were calculated using the formula $r = z / \sqrt{n}$, where 'n' is the number of observations, in this case 2 x 129 = 258. Thresholds used for qualitative descriptions of effect size were: zero or nearly zero effect, $0 < |r| \leq 0.1$; small, $0.1 < |r| \leq 0.3$; moderate, $0.3 < |r| \leq 0.5$; large $0.5 < |r| \leq 0.7$; and very large, $|r| > 0.7$. Data were obtained through an online survey among 129 Dutch pest controllers. Importance could be indicated on a 1 (not important) to 10 (very important) interval rating scale.

Table S4: Exact *P* values and effect sizes for importance of animal welfare between animal categories

	**Companion animals (9;10-8)**	**Farm animals (9;10-8)**	**Other wild animals (9;10-8)**	**Laboratory animals (9;10-7)**	**Rats and mice as pests (5; 7-3)**
**Companion animals (9;10-8)**		0.1908	0.2132	0.3665	0.5591
**Farm animals (9;10-8)**	0.001511		0.1187	0.2835	0.5730
**Other wild animals (9;10-8)**	0.000425	0.056446		0.1540	0.5251
**Laboratory animals (9;10-7)**	<0.0000005*	0.000001*	0.012791		0.5248
**Rats and mice as pests (5; 7-3)**	<0.0000005*	<0.0000005*	<0.0000005*	<0.0000005*	

Table S4: Exact (2-tailed) *P* values (under de diagonal line) and absolute effect sizes (above the diagonal line) for differences in the importance of animal welfare for five different animal categories. Exact (2-tailed) *P* values were calculated for each pair of methods with the Wilcoxon matched-pairs signed ranks test for 2 dependent variables. Numbers in parentheses display the median and the interquartile range (Q3-Q1). Significant *P* values are marked with an asterisk (*). Effect sizes (*r*) were calculated using the formula $r = z / \sqrt{n}$, where '*n*' is the number of observations, in this case 2 x 129 = 258. Thresholds used for qualitative descriptions of effect size were: zero or nearly zero effect, $0 < |r| \leq 0.1$; small, $0.1 < |r| \leq 0.3$; moderate, $0.3 < |r| \leq 0.5$; large $0.5 < |r| \leq 0.7$; and very large, $|r| > 0.7$. Data were obtained through an online survey among 129 Dutch pest controllers. Importance could be indicated on a 1 (not important) to 10 (very important) interval rating scale.

Table S5: Exact *P* values and effect sizes for welfare impact of control methods

	**Glue board (10;10-6)**	**Trap and drown (8;10-6)**	**Rodenticides (7;8-4)**	**Eko-1000 (6;8-4)**	**Trap and release (5;8-2)**	**Cat, dog, ferret (5;8-3)**	**CO2 trap (4;7-2)**	**Killing trap (3;7-2)**	**Shoot (3;7-2)**	**Preventive methods (1;3-1)**
**Glue board (10;10-6)**		0.1019	0.3690	0.3687	0.3795	0.4761	0.4760	0.5153	0.5340	0.5684
**Trap and drown (8;10-6)**	0.102432		0.2980	0.3628	0.3391	0.4496	0.4732	0.4837	0.4875	0.5352
**Rodenticides (7;8-4)**	<0.0000005*	<0.0000005*		0.0766	0.1591	0.1829	0.4007	0.3787	0.4153	0.5370
**Eko-1000 (6;8-4)**	<0.0000005*	<0.0000005*	0.220169		0.0885	0.1101	0.3011	0.3186	0.3398	0.4954
**Trap and release (5;8-2)**	<0.0000005*	<0.0000005*	0.010254	0.155825		0.0043	0.1680	0.1796	0.2122	0.4425
**Cat, dog, ferret (5;8-3)**	<0.0000005*	<0.0000005*	0.003023	0.077099	0.945655		0.2021	0.2254	0.3060	0.4796
**CO2 trap (4;7-2)**	<0.0000005*	<0.0000005*	<0.0000005*	<0.0000005*	0.006603	0.001007		0.0196	0.0550	0.3837
**Killing trap (3;7-2)**	<0.0000005*	<0.0000005*	<0.0000005*	<0.0000005*	0.003667	0.00023	0.755426		0.0519	0.3960
**Shoot (3;7-2)**	<0.0000005*	<0.0000005*	<0.0000005*	<0.0000005*	0.000552	<0.0000005*	0.37898	0.407739		0.3467
**Preventive methods (1;3-1)**	<0.0000005*	<0.0000005*	<0.0000005*	<0.0000005*	<0.0000005*	<0.0000005*	<0.0000005*	<0.0000005*	<0.0000005*	

Table S5: Exact (2-tailed) *P* values (under de diagonal line) and absolute effect sizes (above the diagonal line) for differences in the welfare impact of methods for the control of rats (*Rattus rattus* and *Rattus norvegicus*) and mice (*Mus musculus*). Exact (2-tailed) *P* values were calculated for each pair of methods with the Wilcoxon matched-pairs signed ranks test for 2 dependent variables. Numbers in parentheses display the median and the interquartile range (Q3-Q1). Significant *P* values are marked with an asterisk (*). Effect sizes (*r*) were calculated using the formula $r = z / \sqrt{n}$, where '*n*' is the number of observations, in this case 2 x 129 = 258. Thresholds used for qualitative descriptions of effect size were: zero or nearly zero effect, $0 < |r| \leq 0.1$; small, $0.1 < |r| \leq 0.3$; moderate, $0.3 < |r| \leq 0.5$; large $0.5 < |r| \leq 0.7$; and very large, $|r| > 0.7$. Data were obtained through an online survey among 129 Dutch pest controllers. Welfare impact could be scored on a 1 (no impact) to 10 (very large impact) interval rating scale.

Table S6: Exact *P* values and effect sizes for weight of animal interest for different scenarios

	Rats in a ditch (8;10-6)	Rats along a golf course (8;9-5)	Mice in a backyard (7;9-5)	Rats in the sewers (7;9-5)	Rats in a backyard (5;7-3)	Rats at a garbage plant (5;7-3)	Mice in an animal shelter (5;7-3)	Rats in a cow stable (5;6-2)	Mice on a pig farm (5;6-2)	Rats on a children's farm (3;6-1)	Mice in a supermarket (2;4-1)	Mice in a hospital kitchen (1;3-1)
**Rats in a ditch (8;10-6)**		0.1425	0.2517	0.2279	0.4610	0.4753	0.5022	0.5060	0.5138	0.5383	0.5447	0.5676
**Rats along a golf course (8;9-5)**	0.02161		0.1256	0.1074	0.4259	0.4291	0.4710	0.4652	0.4651	0.5168	0.5309	0.5548
**Mice in a backyard (7;9-5)**	0.000031	0.043337		0.0090	0.4236	0.3818	0.4306	0.4753	0.4859	0.5027	0.5057	0.5474
**Rats in the sewers (7;9-5)**	0.000187	0.08475	0.886849		0.3168	0.3099	0.3990	0.4235	0.4169	0.4687	0.4857	0.5388
**Rats in a backyard (5;7-3)**	<0.0000005*	<0.0000005*	<0.0000005*	<0.0000005*		0.0100	0.1569	0.2113	0.2212	0.3874	0.4083	0.5012
**Rats at a garbage plant (5;7-3)**	<0.0000005*	<0.0000005*	<0.0000005*	<0.0000005*	0.873197		0.1695	0.2220	0.2366	0.3529	0.4202	0.4907
**Mice in an animal shelter (5;7-3)**	<0.0000005*	<0.0000005*	0.01126	<0.0000005*	<0.0000005*	0.006075		0.0584	0.0689	0.2928	0.3639	0.4784
**Rats in a cow stable (5;6-2)**	<0.0000005*	<0.0000005*	<0.0000005*	<0.0000005*	0.000568	0.000271	0.351563		0.0300	0.2157	0.3488	0.4584
**Mice on a pig farm (5;6-2)**	<0.0000005*	<0.0000005*	<0.0000005*	<0.0000005*	0.000299	0.000097	0.270805	0.642867		0.2048	0.3442	0.4550
**Rats on a children's farm (3;6-1)**	<0.0000005*	<0.0000005*	<0.0000005*	<0.0000005*	<0.0000005*	<0.0000005*	<0.0000005*	0.000414	0.000836		0.1494	0.3657
**Mice in a supermarket (2;4-1)**	<0.0000005*	<0.0000005*	<0.0000005*	<0.0000005*	<0.0000005*	<0.0000005*	<0.0000005*	<0.0000005*	<0.0000005*	0.015788		0.2384
**Mice in a hospital kitchen (1;3-1)**	<0.0000005*	<0.0000005*	<0.0000005*	<0.0000005*	<0.0000005*	<0.0000005*	<0.0000005*	<0.0000005*	<0.0000005*	<0.0000005*	0.000072	

Table S6: Exact (2-tailed) *P* values (under de diagonal line) and absolute effect sizes (above the diagonal line) for differences in the weight of animal interests for different real-life scenarios of the control of rats (*Rattus rattus* and *Rattus norvegicus*) and mice (*Mus musculus*). Animal interests were defined as 'living, freedom and welfare'. Exact (2-tailed) *P* values were calculated for each pair of methods with the Wilcoxon matched-pairs signed ranks test for 2 dependent variables. Numbers in parentheses display the median and the interquartile range (Q3-Q1). Significant *P* values are marked with an asterisk (*). Effect sizes (r) were calculated using the formula $r = z / \sqrt{n}$, where 'n' is the number of observations, in this case 2 x 129 = 258. Thresholds used for qualitative descriptions of effect size were: zero or nearly zero effect, $0 < |r| \leq 0.1$; small, $0.1 < |r| \leq 0.3$; moderate, $0.3 < |r| \leq 0.5$; large $0.5 < |r| \leq 0.7$; and very large, $|r| > 0.7$. Data were obtained through an online survey among 129 Dutch pest controllers. Weight of animal interests (e.g. welfare) could be scored on a 1 (animal interests do not weigh) to 10 (animal interests weigh heavily) interval rating scale.

Table S7: Exact *P* values and effect sizes for client investments in prevention

	**Food industry (8;9-7)**	**Health care (8;9-7)**	**Supermarkets (6;8-4)**	**Animal shelters / zoo's (6;7-5)**	**Bakery, butchery, etc. (6;7-4)**	**Restaurants, bars, etc. (5;6-3)**	**Private persons (4;7-2)**	**Municipalities (4;6-3)**	**Garbage processing (4;6-2)**	**Agricultural sector (4;6-2)**
**Food industry (8;9-7)**		0.0574	0.4165	0.4511	0.4600	0.5641	0.5060	0.5490	0.5305	0.5548
**Health care (8;9-7)**	0.406555		0.4341	0.3980	0.4382	0.5122	0.5071	0.5290	0.4996	0.5174
**Supermarkets (6; 8-4)**	<0.0000005*	<0.0000005*		0.0374	0.1583	0.3815	0.3015	0.3801	0.3893	0.4435
**Animal shelters / zoo's (6;7-5)**	<0.0000005*	<0.0000005*	0.618869		0.0168	0.2610	0.2562	0.3479	0.3712	0.4359
**Bakery, butchery, etc. (6;7-4)**	<0.0000005*	<0.0000005*	0.015664	0.821782		0.3194	0.2268	0.3329	0.3661	0.3973
**Restaurants, bars, etc. (5;6-3)**	<0.0000005*	<0.0000005*	<0.0000005*	0.000292	<0.0000005*		0.0088	0.0946	0.2012	0.2452
**Private persons (4;7-2)**	<0.0000005*	<0.0000005*	0.000003*	0.000387	0.000364	0.892706		0.0066	0.1128	0.1076
**Municipalities (4;6-3)**	<0.0000005*	<0.0000005*	<0.0000005*	<0.0000005*	<0.0000005*	0.159846	0.922022		0.1387	0.1370
**Garbage processing (4;6-2)**	<0.0000005*	<0.0000005*	<0.0000005*	<0.0000005*	<0.0000005*	0.004246	0.111241	0.053165		0.0451
**Agricultural sector (4;6-2)**	<0.0000005*	<0.0000005*	<0.0000005*	<0.0000005*	<0.0000005*	0.000284	0.116006	0.052529	0.54103	

Table S7: Exact (2-tailed) *P* values (under de diagonal line) and absolute effect sizes (above the diagonal line) for differences in the willingness to invest in preventive methods among clients according to Dutch pest controllers. Exact (2-tailed) *P* values were calculated for each pair of methods with the Wilcoxon matched-pairs signed ranks test for 2 dependent variables. Numbers in parentheses display the median and the interquartile range (Q3-Q1). Significant *P* values are marked with an asterisk (*). Effect sizes (*r*) were calculated using the formula $r = z / \sqrt{n}$, where '*n*' is the number of observations, in this case 2 x 129 = 258. Thresholds used for qualitative descriptions of effect size were: zero or nearly zero effect, $0 < |r| \leq 0.1$; small, $0.1 < |r| \leq 0.3$; moderate, $0.3 < |r| \leq 0.5$; large $0.5 < |r| \leq 0.7$; and very large, $|r| > 0.7$. Data were obtained through an online survey among 129 Dutch pest controllers. Willingness to invest in preventive methods could be scored on a 1 (no willingness to invest) to 10 (much willingness to invest) interval rating scale.

Table S8: Exact *P* values and effect sizes for Solutions for problems in practice

	**More client willingness for prevention (9;10-8)**	**More client awareness through education (8;10-7)**	**Governmental subsidies for preventive methods (8;9-6)**	**More attention for dilemma's in education (7;9-6)**	**Adjustment of regulations/laws (6;8-4)**	**Certification systems for pest controllers (6;9-3)**	**A decision tree for pest controllers (6;8-3)**
**More client willingness for prevention (9;10-8)**		0.1535	0.3121	0.3806	0.5174	0.4845	0.5220
**More client awareness through education (8;10-7)**	0.013086		0.1839	0.3020	0.4585	0.4177	0.5005
**Governmental subsidies for preventive methods (8;9-6)**	<0.0000005*	0.002842		0.1088	0.2810	0.2563	0.3813
**More attention for dilemma's in education (7;9-6)**	<0.0000005*	<0.0000005*	0.080809		0.2890	0.2682	0.4092
**Adjustment of regulations/laws (6;8-4)**	<0.0000005*	<0.0000005*	0.000003*	0.000001*		0.0024	0.1476
**Certification systems for pest controllers (6;9-3)**	<0.0000005*	<0.0000005*	0.000025*	0.000009*	0.971056		0.1294
**A decision tree for pest controllers (6;8-3)**	<0.0000005*	<0.0000005*	<0.0000005*	<0.0000005*	0.017259	0.037329	

Table S8: Exact (2-tailed) *P* values (under de diagonal line) and absolute effect sizes (above the diagonal line) for differences in the added value of possible solutions to overcome problems in rodent control daily practice according to Dutch pest controllers. Exact (2-tailed) *P* values were calculated for each pair of methods with the Wilcoxon matched-pairs signed ranks test for 2 dependent variables. Numbers in parentheses display the median and the interquartile range (Q3-Q1). Significant *P* values are marked with an asterisk (*). Effect sizes (*r*) were calculated using the formula $r = z / \sqrt{n}$, where '*n*' is the number of observations, in this case 2 x 129 = 258. Thresholds used for qualitative descriptions of effect size were: zero or nearly zero effect, $0 < |r| \leq 0.1$; small, $0.1 < |r| \leq 0.3$; moderate, $0.3 < |r| \leq 0.5$; large $0.5 < |r| \leq 0.7$; and very large, $|r| > 0.7$. Data were obtained through an online survey among 129 Dutch pest controllers. Added value of the possible solutions could be scored on a 1 (no added value) to 10 (large added value) interval rating scale.

Table S9: Exact *P* values and effect sizes for Work motivation

	**Solve problems for clients (10;10-9)**	**Contribute to food safety (10;10-9)**	**Contribute to safe and healthy environment (9;10-8)**	**Prevent economic losses (9;10-8)**	**Contribute to nature conservation (9;10-7)**	**Guarantee income (8;10-7)**
**Solve problems for clients (10;10-9)**		0.0278	0.2459	0.2932	0.3332	0.4074
**Contribute to food safety (10;10-9)**	0.661678		0.2776	0.3351	0.3563	0.4059
**Contribute to safe and healthy environment (9;10-8)**	0.000042*	0.000002*		0.0544	0.2219	0.2639
**Prevent economic losses (9;10-8)**	<0.0000005*	<0.0000005*	0.382457		0.1315	0.2470
**Contribute to nature conservation (9;10-7)**	<0.0000005*	<0.0000005*	0.000242	0.034225		0.1020
**Guarantee income (8;10-7)**	<0.0000005*	<0.0000005*	0.000012*	0.000046*	0.101712	

Table S9: Exact (2-tailed) *P* values (under de diagonal line) and absolute effect sizes (above the diagonal line) for differences between aspects of work motivation according to Dutch pest controllers. Exact (2-tailed) *P* values were calculated for each pair of methods with the Wilcoxon matched-pairs signed ranks test for 2 dependent variables. Numbers in parentheses display the median and the interquartile range (Q3-Q1). Significant *P* values are marked with an asterisk (*). Effect sizes (*r*) were calculated using the formula $r = z / \sqrt{n}$, where '*n*' is the number of observations, in this case 2 x 129 = 258. Thresholds used for qualitative descriptions of effect size were: zero or nearly zero effect, $0 < |r| \leq 0.1$; small, $0.1 < |r| \leq 0.3$; moderate, $0.3 < |r| \leq 0.5$; large $0.5 < |r| \leq 0.7$; and very large, $|r| > 0.7$. Data were obtained through an online survey among 129 Dutch pest controllers. Aspects of work motivation could be scored on a 1 (not important) to 10 (very important) interval rating scale.

Table S10: Exact *P* values and effect sizes of independent samples

	PLA..N. *vs.* NVPB	PLA..N. vs. Unspecified member	PLA..N. vs. No member	NVPB *vs.* Unspecified member	NVPB *vs.* No member	Unspecified member vs. No member
General attitude of IPM						
F. IPM as a prerequisite for the use of rodenticides for rat control outsite buildings is a good thing						
Exact *P* value	0.000023*	0.55058	0.735457	0.007181	0.000006*	0.545877
Effect size |r|	0.4751	0.0789	0.0395	0.3616	0.5222	0.0835
G. IPM should be a prerequisite for each form of pest control						
Exact *P* value	0.000031*	0.912914	0.964106	0.004565	0.000067*	0.945481
Effect size |r|	0.4683	0.0149	0.0056	0.3810	0.4644	0.0100
Solutions for problems in practice						
Certification systems for pest controllers						
Exact *P* value	0.000037*	0.389512	0.750606	0.003392	0.000257*	0.487925
Effect size |r|	0.4635	0.1133	0.0372	0.3922	0.4278	0.0958
A decision tree for pest controllers						
Exact *P* value	0.002926	0.039344	0.456957	0.000009*	0.024074	0.009739
Effect size |r|	0.3398	0.2678	0.0866	0.5008	0.2688	0.3482

Table S10: Exact (2-tailed) *P* values and absolute effect sizes for differences in attitude of IPM and the added value of possible solutions to overcome problems in rodent control daily practice according to Dutch pest controllers. Exact (2-tailed) *P* values were calculated for each pair of methods with the Wilcoxon-Mann-Whitney test for 2 independent variables. Statistically significant differences between two types of membership (PLA..N.: $n = 40$; NVPB: $n = 35$; Unspecified member: $n = 19$; No member: $n = 35$) are indicated with *. Effect sizes (r) were calculated using the formula $r = z / \sqrt{n}$, where 'n' is the number of observations. Thresholds used for qualitative descriptions of effect size were: zero or nearly zero effect, $0 < |r| \leq 0.1$; small, $0.1 < |r| \leq 0.3$; moderate, $0.3 < |r| \leq 0.5$; large $0.5 < |r| \leq 0.7$; and very large, $|r| > 0.7$. Data were obtained through an online survey among 129 Dutch pest controllers. Added value of the possible solutions could be scored on a 1 (no added value) to 10 (large added value) interval rating scale.
